# Evolution of Toll, Spatzle and MyD88 in insects: the problem of the Diptera bias

**DOI:** 10.1186/s12864-021-07886-7

**Published:** 2021-07-21

**Authors:** Letícia Ferreira Lima, André Quintanilha Torres, Rodrigo Jardim, Rafael Dias Mesquita, Renata Schama

**Affiliations:** 1 Laboratório de Biologia Computacional e Sistemas, Oswaldo Cruz Foundation, Fiocruz, Rio de Janeiro, Brazil; 2 Laboratório de Bioinformática, Instituto de Química, Universidade Federal do Rio de Janeiro, Rio de Janeiro, Brazil; 3 Instituto Nacional de Ciência e Tecnologia em Entomologia Molecular-INCT-EM, Rio de Janeiro, Brazil

**Keywords:** Arthropoda, Evolution, Gene family, Innate immunity, Hexapoda, Pelle, Pellino, Tube, Toll pathway, SSN

## Abstract

**Background:**

Arthropoda, the most numerous and diverse metazoan phylum, has species in many habitats where they encounter various microorganisms and, as a result, mechanisms for pathogen recognition and elimination have evolved. The Toll pathway, involved in the innate immune system, was first described as part of the developmental pathway for dorsal-ventral differentiation in *Drosophila*. Its later discovery in vertebrates suggested that this system was extremely conserved. However, there is variation in presence/absence, copy number and sequence divergence in various genes along the pathway. As most studies have only focused on Diptera, for a comprehensive and accurate homology-based approach it is important to understand gene function in a number of different species and, in a group as diverse as insects, the use of species belonging to different taxonomic groups is essential.

**Results:**

We evaluated the diversity of Toll pathway gene families in 39 Arthropod genomes, encompassing 13 different Insect Orders. Through computational methods, we shed some light into the evolution and functional annotation of protein families involved in the Toll pathway innate immune response. Our data indicates that: 1) intracellular proteins of the Toll pathway show mostly species-specific expansions; 2) the different Toll subfamilies seem to have distinct evolutionary backgrounds; 3) patterns of gene expansion observed in the Toll phylogenetic tree indicate that homology based methods of functional inference might not be accurate for some subfamilies; 4) Spatzle subfamilies are highly divergent and also pose a problem for homology based inference; 5) Spatzle subfamilies should not be analyzed together in the same phylogenetic framework; 6) network analyses seem to be a good first step in inferring functional groups in these cases. We specifically show that understanding *Drosophila*’s Toll functions might not indicate the same function in other species.

**Conclusions:**

Our results show the importance of using species representing the different orders to better understand insect gene content, origin and evolution. More specifically, in intracellular Toll pathway gene families the presence of orthologues has important implications for homology based functional inference. Also, the different evolutionary backgrounds of Toll gene subfamilies should be taken into consideration when functional studies are performed, especially for TOLL9, TOLL, TOLL2_7, and the new TOLL10 clade. The presence of Diptera specific clades or the ones lacking Diptera species show the importance of overcoming the Diptera bias when performing functional characterization of Toll pathways.

**Supplementary Information:**

The online version contains supplementary material available at 10.1186/s12864-021-07886-7.

## Background

Arthropoda is the most numerous and diverse metazoan phylum [1–4]. It is an extremely successful group, with species present in almost all habitats on earth. Insects alone account for more than 1 million species that have a wide spectrum of adaptations [1]. Given their abundance, evolutionary resilience and widespread presence, many insect species importantly impact human health [5]. Many are vectors of pathogens and others are pests of agricultural or metropolitan importance [5–7]. Pollinators and other species responsible for recycling dead matter are also of significant importance in a One Health perspective [8, 9]. Insect presence in most habitats, with their wide variety of dietary habits and behavior, also means that they encounter various microorganisms such as bacteria, fungi and viruses, many of which may be pathogenic. As a result, insects have evolved mechanisms for pathogen recognition and elimination [10–12]. Although it is not clear if insects have some type of adaptive immune response [13–16], cellular and humoral responses against pathogens have been well characterized [10, 17–19].

Innate immunity is the first line of defense that controls the initial steps of the immune response in multicellular organisms [11, 20–24]. In insects, four different immune signaling pathways have been described: Imd, Toll, JAK/STAT and RNAi [21, 25]. The RNAi pathway mainly controls virus replication [26] while the JAK/STAT pathway regulates immune response genes related to viral and bacterial infections. The Imd and Toll pathways are inflammatory responses that include the recognition of pathogens and expression of a wide spectrum of anti-microbial peptides (AMPs) through the activation of NF-kB-like (Nuclear Factor-kappa B-like) transcription factors [27–30]. Both signal transduction pathways link the recognition of pathogen-associated molecular patterns (PAMPs) by Pathogen Recognition Receptors (PRRs) with transcriptional activation [31–35]. The Toll pathway has first been described as part of the developmental pathway for dorsal-ventral differentiation in *Drosophila* [36, 37]. Since then, the many gene families involved in the different Toll pathways have been shown to be important not only for immune response but for all kinds of inflammatory and non-inflammatory responses even without pathogen presence [29, 38]. Although previously this pathway has only been linked to defense against gram-positive bacteria and fungi, more recently, in *Drosophila*, many different functions and pathways have been discovered where Toll genes are essential.

In the fruit fly, it has been demonstrated that Toll signal transduction initiates when a cleaved protein dimer ligand binds to the extracellular domain of Toll receptors [39–42]. Conventionally, a phosphorylation cascade then initiates with the intracellular domain of Toll binding to another transmembrane protein, MyD88 [43–46]. Subsequently, MyD88 forms an heterotrimer with the scaffolding protein Tube and Pelle (a protein kinase) through their death domains (DD), initiating the signal transduction pathway [47, 48]. With Pellino’s positive regulation of Pelle [49], this complex phosphorylates Cactus which releases Dorsal or Dif (Dorsal-related immunity factor), both members of the Rel family of transcription factors, which translocate into the nucleus activating different genes, including antimicrobial ones such as the antifungal peptide Drosomycin, for example [10, 48, 50, 51].

Toll-like receptors (TLRs) are a family of type I transmembrane proteins with an ectodomain composed of repeats of leucine-rich regions (LRRs) flanked by cysteine-rich modules and an intracytoplasmic signaling TIR domain (a Toll/interleukin-1 receptor domain homologue) [51–56]. To date, nine genes have been found in *Drosophila melanogaster*’s genome and similar numbers were found in other insects [51, 57–60]. Although in humans Toll-like receptors act in pathogen recognition, in insects, Toll functions more like cytokine receptors, mostly for the endogenous protein Spatzle (Spz) [54, 61–64]. Spatzle was also originally identified as a component of the dorsal-ventral patterning signaling pathway that acts upstream of Toll. Since then, other five Spatzle homologues (Spz2–6) have been identified in *Drosophila* [55]. All of them encode extracellular proteins with neurotrophin-like cysteine-knot domains. Spatzle is activated by protease cleavage [65] and its C-terminal fragment is believed to be the one to bind to the extracellular domain of Toll and activate its pathway [63, 66]. Upon cleavage, the Spatzle fragments form a dimer held together by intermolecular disulphide bridges [42]. In the embryo, precise spatial regulation of Spatzle activation is necessary for normal dorsal-ventral development but in larval and adult stages both Spatzle and its upstream activating proteases are openly circulating in the hemolymph [67, 68]. The precise mechanisms by which Spatzle is recognized and activated and how this leads to which Toll pathway is activated is not completely clear. In *Drosophila*, danger signals and Damage Associated Molecular Patterns (DAMPs) may also activate Persephone, one of the proteases responsible for cleaving Spatzle [38, 69, 70]. This response seems important in differentiating harmful microbes from commensal ones.

The finding of Toll-like structures in vertebrates led to the belief that the innate immune system was extremely conserved. Nevertheless, although very similar in structure and pathway formation, vertebrate and most Arthropod Toll genes seem to be associated with two unrelated events of gene expansion [23, 51]. In arthropods, genes from both Toll and Imd signaling pathways are conserved, with more sequence variation in recognition and effector genes than in those in the middle of the pathway [60, 71, 72]. Nevertheless, there is also variation in presence/absence, copy number and sequence divergence in various genes along the pathway. As more taxonomic groups are investigated, more diversity is found, sometimes with whole pathways missing. In aphids and chelicerates, for example, some or all Imd genes are missing [71, 73].

The fact that most studies have focused on Diptera obscured the knowledge of the significance of these immune system related genes in other insect groups. For a comprehensive and accurate homology-based approach it is important to understand gene function in a number of different species and, in a group as diverse as insects, the use of species belonging to different taxonomic groups is essential. Given the large evolutionary time scales, many lineage specific changes may have occurred. Insects first appeared in the fossil record ~ 412 million years ago (MYA) and it is difficult to predict function from BLAST searches when comparing species that have diverged hundreds of millions of years ago. The Dipterans, for example, seem to have emerged in the Permian (~ 250 MYA) and the Culicidae genera *Anopheles* and *Aedes* seem to have diverged ~ 170 MYA [1, 74–76]. Also, it has already been demonstrated that in many cases the presence of copy number variation can be accompanied by changes in function [71, 77]. Newly sequenced insect genomes have their genes annotated based on sequence homology to known genes from other species, so it is crucial that homology-based studies are performed so we better understand the different gene duplications in these protein families.

In this study, we analyze 39 insect genomes belonging to 13 insect orders encompassing the three principal Neoptera groups (Polyneoptera, Paraneoptera and Holometabola) and the Palaeoptera (Odonata and Ephemeroptera) [1, 78] together with the Crustacea *Daphnia pulex* to shed some light in the evolution of six gene families of the Toll pathway in Insecta. We focused on genes previously considered to be less diverse and, therefore, less investigated. To our knowledge, this is the first genomic study with so many insect orders to focus specifically on Toll receptors and other gene families involved in the Toll pathway, which encode proteins that interact either directly or indirectly with Toll.

## Results

### Protein searches

Sequences of putative Toll (396), MyD88 (60), Spatzle (1069, of which 476 are unique ones), Tube (55), Pelle (47) and Pellino (75) proteins were identified from the predicted protein sets of 39 insects and from the crustacean *D. pulex.* Table 1 summarizes the organisms analyzed and number of copies of each gene found in each genome and their source. Only in a few cases the automated genome predictions did not contain one or more of the proteins expected for the protein families and subfamilies analyzed and these were, therefore, searched for with Exonerate searches of the scaffolds (see Additional file 1). Incomplete predictions were recovered and the protein was only counted as existent in a species when a significant identity value and good coverage was found with subsequent BLASTp searches. A supplementary text file, in FASTA format, with Transeq translation of proteins recovered with Exonerate is available (see Additional file 2).

**Table 1:** List of species analyzed and number of proteins found for each gene family searched

Subphylum	Order	Family	Species	Database	Version	MyD88	Tube	Pelle	Pellino	Spatzel	Total Toll	Toll 9	Toll, Toll3_4_5	Toll8	Toll6	Toll2_7	Toll10	
Crustacea	Diplostraca	Daphniidae	*Daphnia pulex*	ncbi	V1.0	1	1	1	1	48	5	1	2	1	0	1	0	
Hexapoda	Blattodea	Ectobiidae	*Blattella germanica*	ncbi	Bger_1.1	3	1	1	2	6	6	1	1	1	1	1	1	
Hexapoda	Blattodea	Kalotermitidae	*Cryptotermes secundus*	ncbi	Csec_1.0	2	4	1	5	19	7	2	1	1	1	1	1	
Hexapoda	Coleoptera	Scarabaeidae	*Onthophagus taurus*	ncbi	Otau_2.0	1	1	1	2	20	12	6	1	1	1	1	2	
Hexapoda	Coleoptera	Tenebrionidae	*Tribolium castaneum*	ncbi	Tcas5.2	3	3	2	3	16	8	1	3	1	1	1	1	
Hexapoda	Diptera	Calliphoridae	*Lucilia cuprina*	ncbi	Lcup_2.0	2	1	1	1	7	9	1	4	1	1	2	0	
Hexapoda	Diptera	Culicidae	*Aedes aegypti*	ncbi	AaegL5.0	1	2	1	4	15	16	3	7	1	2	1	2	
Hexapoda	Diptera	Culicidae	*Anopheles funestos*	vectorbase	AfunF1.8	1	1	1	1	6	9	1	3	1	1	1	2	
Hexapoda	Diptera	Culicidae	*Anopheles gambiae*	ncbi	AgamP3	1	1	1	2	7	13	1	7	1	1	1	2	
Hexapoda	Diptera	Culicidae	*Culex quinquefasciatus*	ncbi	CpipJ2.4	2	1	1	1	7	8	2	1	2	1	1	1	
Hexapoda	Diptera	Drosophilidae	*Drosophila ananassae*	ncbi	dana_caf1	1	1	3	1	19	12	3	5	1	1	2	0	
Hexapoda	Diptera	Drosophilidae	*Drosophila melanogaster*	ncbi	Release 6 plus ISO1 MT	3	1	2	2	14	16	3	7	1	3	2	0	
Hexapoda	Diptera	Drosophilidae	*Drosophila willistoni*	flybase	dwil_r1.05_FB2016_05	1	1	1	1	9	12	1	7	1	1	2	0	
Hexapoda	Diptera	Glossinidae	*Glossina brevipalpis*	vectorbase	GbreI1.6	1	1	1	1	7	7	1	2	1	1	2	0	
Hexapoda	Diptera	Glossinidae	*Glossina fuscipes*	vectorbase	GfusI1.6	1	1	1	1	9	6	1	1	1	1	2	0	
Hexapoda	Diptera	Muscidae	*Musca domestica*	ncbi	Musca_domestica_.0.2	1	1	1	1	8	6	1	1	1	1	2	0	
Hexapoda	Diptera	Muscidae	*Stomoxys calcitrans*	vectorbase	ScalU1.4	1	1	1	3	6	8	2	1	1	1	2	0	
Hexapoda	Diptera	Psychodidae	*Lutzomyia longipalpis*	vectorbase	LlonJ1.5	1	1	1	1	4	6	1	1	1	1	2	0	
Hexapoda	Diptera	Psychodidae	*Phlebotomus papatasi*	vectorbase	PpapI1.4	2	1	0	1	3	5	1	1	1	1	1	0	
Hexapoda	Diptera	Tephritidae	*Bactrocera dorsalis*	ncbi	ASM78921v2	2	1	2	2	17	13	1	8	1	1	2	0	
Hexapoda	Diptera	Tephritidae	*Ceratitis capitata*	ncbi	Ccap_2.1	1	1	2	1	17	14	2	7	1	1	3	0	
Hexapoda	Diptera	Tephritidae	*Rhagoletis zephyria*	ncbi	Rhagoletis_zephyria_1.0	1	1	0	1	19	18	3	10	1	1	3	0	
Hexapoda	Ephemeroptera	Ephemeridae	*Ephemera danica*	i5k.nal	02-Mar-2018 15:27	1	1	1	1	7	22	16	1	1	1	2	1	
Hexapoda	Hemiptera	Aphididae	*Acyrthosiphon pisum*	ncbi	Acyr_2.0	3	3	1	1	13	8	1	3	1	1	1	1	
Hexapoda	Hemiptera	Reduviidae	*Rhodnius prolixus*	vectorbase	RproC3.3	2	1	1	1	4	6	1	1	1	1	1	1	
Hexapoda	Hymenoptera	Apidae	*Apis mellifera*	ncbi	Amel_HAv3.1	2	1	2	2	10	10	0	6	1	1	1	1	
Hexapoda	Hymenoptera	Apidae	*Bombus impatiens*	ncbi	BIMP_2.1	1	1	0	5	14	7	0	2	1	1	1	2	
Hexapoda	Hymenoptera	Formicidae	*Acromyrmex echinatior*	ncbi	Aech_3.9	3	1	0	3	15	5	0	1	1	1	1	1	
Hexapoda	Hymenoptera	Formicidea	*Camponotus floridanus*	ncbi	Cflo_v7.5	1	1	1	2	14	5	0	1	1	1	1	1	
Hexapoda	Hymenoptera	Megachilidae	*Megachile rotundata*	ncbi	MROT_1.0	2	5	0	5	12	8	0	2	1	2	2	1	
Hexapoda	Hymenoptera	Pteromalidae	*Nasonia vitripennis*	ncbi	Nvit_2.1	1	2	6	1	12	9	0	5	1	1	1	1	
Hexapoda	Lepidoptera	Bombycidae	*Bombyx mori*	ncbi	ASM15162v1	2	1	1	1	16	12	2	3	1	1	3	2	
Hexapoda	Lepidoptera	Nymphalidae	*Heliconius melpomene*	ensemblgenomes	9-Mar-2018	1	1	1	1	9	10	1	1	1	2	3	2	
Hexapoda	Lepidoptera	Sphingidae	*Manduca sexta*	i5k.nal	02-Sep-2014	1	2	0	4	16	28	4	11	2	2	5	4	
Hexapoda	Odonata	Libellulidae	*Ladona fulva*	i5k.nal	02-Mar-2018 1	1	1	1	2	8	7	3	1	1	1	1	0	
Hexapoda	Orthoptera	Acrididae	*Locusta migratoria*	i5k.nal	09-May-2017 15:21	1	1	1	1	6	13	5	4	1	1	1	1	
Hexapoda	Phtiraptera	Pediculidae	*Pediculus humanus*	vectorbase	PhumU2.4	1	1	1	1	8	6	1	1	1	1	1	1	
Hexapoda	Siphonaptera	Pulicidae	*Ctenocephalides felis*	ncbi	ASM342690v1	1	1	2	1	11	9	1	1	0	1	5	1	
Hexapoda	Thysanoptera	Thripidae	*Frankliniella occidentalis*	ncbi	Focc_2.1	2	2	2	4	13	8	3	1	1	1	1	1	
Hexapoda	Trichoptera	Lymnephilidae	*Limnephilus lunatus*	i5k.nal	19-Mar-2015	1	1	0	1	5	5	0	1	1	1	1	1	

Among the Toll subfamilies, Toll9 genes were not found in the six Hymenoptera species analyzed and the only Trichoptera genome searched, suggesting that this subfamily was lost in these lineages. Nevertheless, since we only have one Trichoptera species in our study, problems in the genome assembly should not be ruled out either. Small or partially predicted proteins for the species *Lutzomyia longipalpis*, *Phlebotomus papatasi*, *Glossina brevipalpis* and *Acyrthosiphon pisum*, possibly belonging to the Toll9 subfamily, were found with Exonerate. Although they were counted as Toll9 they were not used in the phylogenetic analysis due to their incomplete prediction (see Additional file 1). For the Toll8 subfamily, one possible gene for the species *Stomoxys calcitrans* was found but reliable predictions could not be made for the species *Ctenocephalides felis*. For Toll6, one possible gene was found for the species *C. felis*, *Locusta migratoria*, *Rhodnius prolixus*, *Bactrocera dorsalis* and two partial predictions were found for *Heliconius melpomene*. No genes were found for *D. pulex* in this subfamily. For the Toll2_7 subfamily, new partially predicted genes were found for *D. pulex*, *Ladona fulva* and *L. migratoria* (see Additional file 1). For the new Toll10 subfamily, no genes were found for the species *D. pulex* and *L. fulva*, but partials were found for *Megachile rotundata*, *Nasonia vitripennis*, *L. migratoria* and *C. felis*. No gene for this subfamily was found in *L. fulva* and *D. pulex*. In Diptera, Toll10 genes were only found in the Culicidae while none were present in the Neodiptera (Schizophora) and Psychodidae species, suggesting it was lost in these two lineages.

Although searched for, the protein Pelle was also not found in the protein sets or with Exonerate searches of the genomes of the species *Rhagoletis zephyria, Phlebotomus papatasi, Megachile rotunda, Bombus impatiens, Acromyrmex echinatior, Manduca sexta* and *Limnephilus lunatus.* Since what differentiates Pelle from other ATP binding proteins is the presence of its Death Domain (DD) and lack of other protein kinase domains, we only included genes that had at least a partial DD together with a protein kinase (Pkinase) domain and no other. In this case, it might be possible that poorly predicted genome regions might have been the cause of gene absence in these species, especially because, apart from Trichoptera, in all other cases other species of the same order did have the gene (Table 1). For MyD88, in addition to the 10 genes recovered with Exonerate (see Additional file 1), we were able to retrieve complete protein sequences for the species *Cryptotermes secundus* (XP_023725093.1, XP_023725092_1), *Stomoxys calcitrans* (XP_013115653_1) and *Bombyx mori* (XP_004921573_1) with BLASTp searches in the GenBank database, even though these were not present in their genome’s protein sets and not found with Exonerate searches. Two new Tube genes were found for the species *Blattella germanica* and *Limnephilus lunatus* and only one Pellino gene for *Limnephilus lunatus* was found. Twenty-one new putative Spatzle proteins were found with Exonerate searches (see Additional file 1).

A few proteins found on the HMMsearches and most of the new genes found with Exonerate were not completely predicted and, therefore, were not used in a phylogenetic context. Nevertheless, they were used in the Sequence Similarity Network analyses and counted as present in the genomes in Table 1. With this approach it was possible to count all genes with the expected domains within the genomes analyzed but still have reliable phylogenetic inferences.

### Sequence similarity networks

Unlike phylogenies, SSNs do not infer evolutionary relationships but demonstrate groups of similar sequences which, together with other sequence information, might suggest similar function or another trend [79–81]. We used SSNs to better understand the different functional groups present in the proteins that have the TIR and Spatzle domains. For the TIR domain, the network contains all sequences retrieved with the HMMsearches and includes edges with an alignment score cut off of 20. This separates the proteins identified as Toll from MyD88, which form separate clusters (see Additional file 3). Toll proteins form two clusters with the smaller one containing Toll sequences that are similar to interleukin-1 receptors and sequences with partial TIR domain and that, therefore, were not used in the phylogenetic analysis (TOLL 2, (see Additional file 3)). Two nodes in grey are outliers and have not formed edges with any other node even though a low stringency SSN was created. These sequences (GBRI043149-PA and XP_026472669.1) were similar to SAP30 and zinc finger genes on BLASTp searches and were retrieved by FAT but do not have a complete TIR-like domain. Sequence identity varied from 25 to 100% and the median for all Toll genes was 34.48% and MyD88 36.88%. A higher stringency network was created to better understand the functional groups within Toll proteins (see Additional file 4). In this case, an alignment score of 20 was used to create the network and, in Cytoscape, an identity value of 50% was also used as threshold and edges with lower values were deleted from the network. The nodes were colored based on taxonomic groups. This analysis already shows groups of taxa-specific clusters, suggesting lineage specific expansions (this is better visualized in the phylogenetic analysis below).

**Fig. 1:**
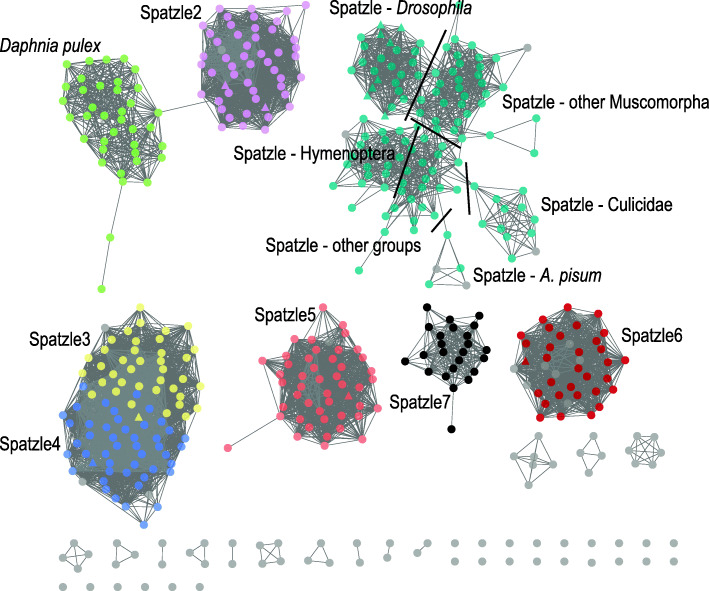
SSN of the Spatzle domain proteins found on FAT searches. Each node represents proteins sharing 100% sequence similarity and edges with an alignment score cut-off of 30 between proteins. Clusters are colored based on OrthoMCL, Blast results and the presence of *Drosophila melanogaster*’s Spatzle genes (triangle shaped nodes). Group names were given based on *D. melanogaster*’s gene name. Grey nodes are unidentified sequences

For the SSN of proteins with Spatzle domain (Fig. 1) (see also Additional file 5) an alignment score of 30 was used which formed clusters of sequences with 25–100% sequence identity. The number of different clusters that have no edges with others already suggests low sequence identity among functional groups. The species *Phlebotomus papatasi* and *Anopheles funestos* have the lowest protein number [3] and the highest number is found in *D. pulex* [35]. Seven bigger (more than seven nodes) different functional groups were formed that more or less coincide with the different *D. melanogaster*’s Spatzle proteins identified previously [55] (triangle shaped nodes in Fig. 1 and Additional file 5). One group (light green in Fig. 1) is formed by sequences of uncharacterized proteins of *D. pulex* only. Other *D. pulex* proteins can be found in five isolated nodes, and one node each can also be found in the Spz2, Spz5, Spz6 and Spz7 clusters described below (see Additional file 5). The *D. pulex* cluster has one edge with the Spz2 protein cluster (light pink, Fig. 1). This cluster is composed of proteins from species of almost all insect orders analyzed with Coleoptera, Trichoptera, Ephemeroptera and Orthoptera being the only ones absent. Another cluster contains both Spz3 (yellow) and Spz4 (blue) proteins and even with a higher identity value stringency it is not possible to further differentiate these two groups. The cluster contains proteins from all insect orders analyzed that fall on both Spz3 and Spz4 regions, however, only one node of Orthoptera proteins is formed. Another cluster is formed by Spz5 sequences (orange) with all insect orders, with the exception of Orthoptera. The cluster of Spz6 proteins (red) contains sequences from all insect orders except Orthoptera and Trichoptera. One smaller cluster, containing non-Diptera uncharacterized proteins (black cluster) from all insect orders except Diptera and Orthoptera was named Spz7. Other smaller clusters, formed mostly by species-specific non-identified sequences and some isolated sequences, are colored grey.

A larger more diverse cluster of Spatzle proteins (cyan) was formed. If we look closely at the clusters within it, we can see five taxa-specific node clusters (Fig. 1 and Additional file 5). One is formed by *Drosophila* species, another by other Schizophora species, a third one contains all Culicidae, the fourth with *A. pisum* sequences and the fifth with Hymenoptera species sequences (see Additional file 5). In the middle, nodes with Siphonaptera, Coleoptera, Blattodea, Orthoptera, Trichoptera, Thysanoptera, Phtiraptera, Psychodidae and the Hemiptera *R. prolixus* sequences are present (see Additional file 5). In Fig. 1, sequences in grey within the different Spatzle clusters did contain a Spatzle domain that were either too small for a confirmation of their orthologous group in OrthoMCL or had other domains attached as well. Due to the high sequence divergence between and within functional groups a phylogenetic analysis was not performed. Phylogenetic analyses of protein sequences with less than 40% sequence identities are not reliable [82], especially when an ancient radiation has happened [83], as is the case for the gene family here. A conservative approach is important due to the possibility of multiple substitutions having occurred at the same site that would not be taken into account in the amino acid substitution model and due to the short internal branches.

### Phylogenetic analyses

**Fig. 2:**
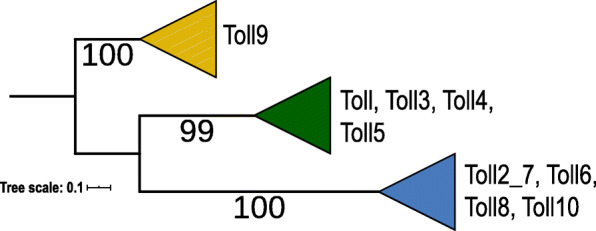
Maximum likelihood phylogeny of the protein alignment of the TIR domain for TOLL sequences. The branches were collapsed for a better visualization of the three main Toll clades. In yellow the Toll9 subclades, in green the clade containing TOLL, TOLL3, TOLL4 and TOLL5 subclades and, in blue, the one containing TOLL2_7, TOLL6, TOLL8 and TOLL10 subclades. Numbers on branches are bootstrap support values from 1000 replicates and only numbers above 50% are shown. Scale bar is substitutions per site. The image was created using iTOL [84]

**Fig. 3:**
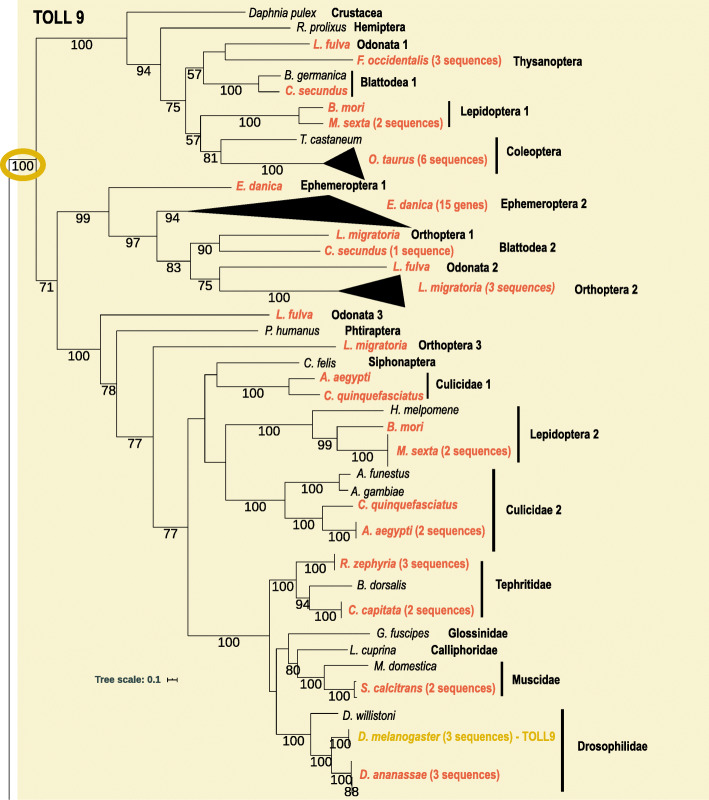
Maximum likelihood phylogeny of the yellow clade of TOLL9 proteins. Species with gene duplications are highlighted in orange and *Drosophila melanogaster*’s Toll9 genes are highlighted on the tree. Numbers on branches are bootstrap support values from 1000 replicates and only numbers above 50% are shown. Scale bar is substitutions per site. The image was created using iTOL [84]

**Fig. 4:**
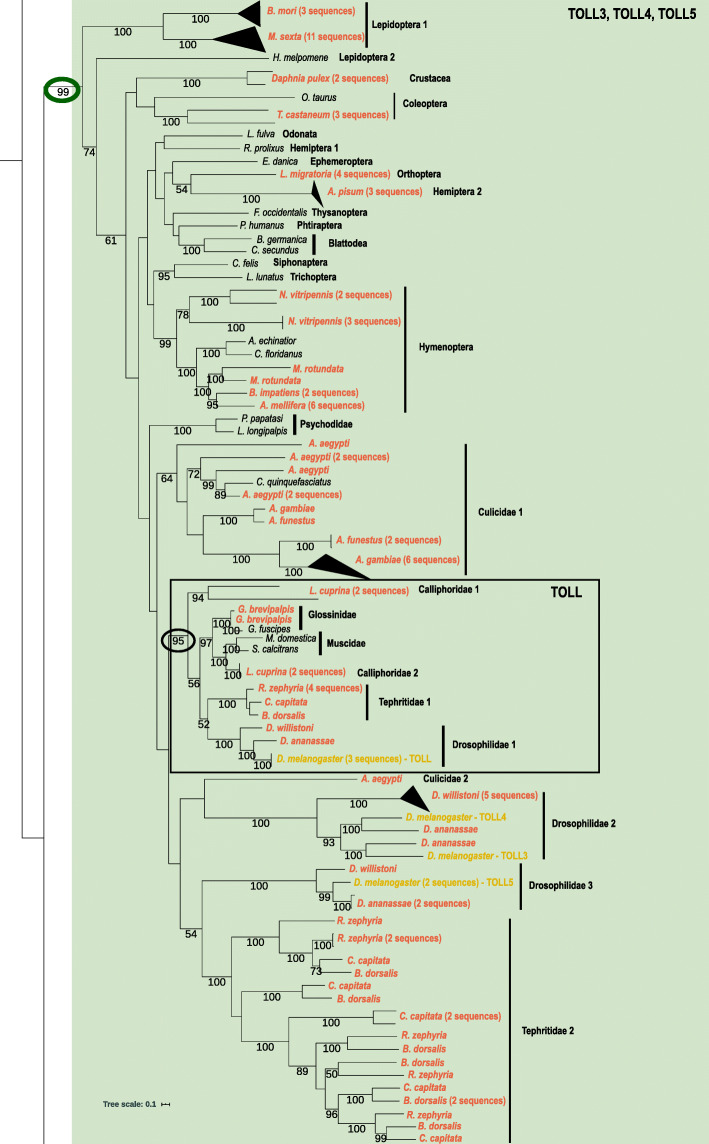
Maximum likelihood phylogeny of the green clade of TOLL, TOLL3, TOLL4 and TOLL5 proteins. Species with gene duplications are highlighted in orange and *Drosophila melanogaster*’s Toll, Toll3, Toll4 and Toll5 genes are highlighted on the tree. The black rectangle highlights the Diptera-specific TOLL clade. Numbers on branches are bootstrap support values from 1000 replicates and only numbers above 50% are shown. Scale bar is substitutions per site. The image was created using iTOL [84]

**Fig. 5:**
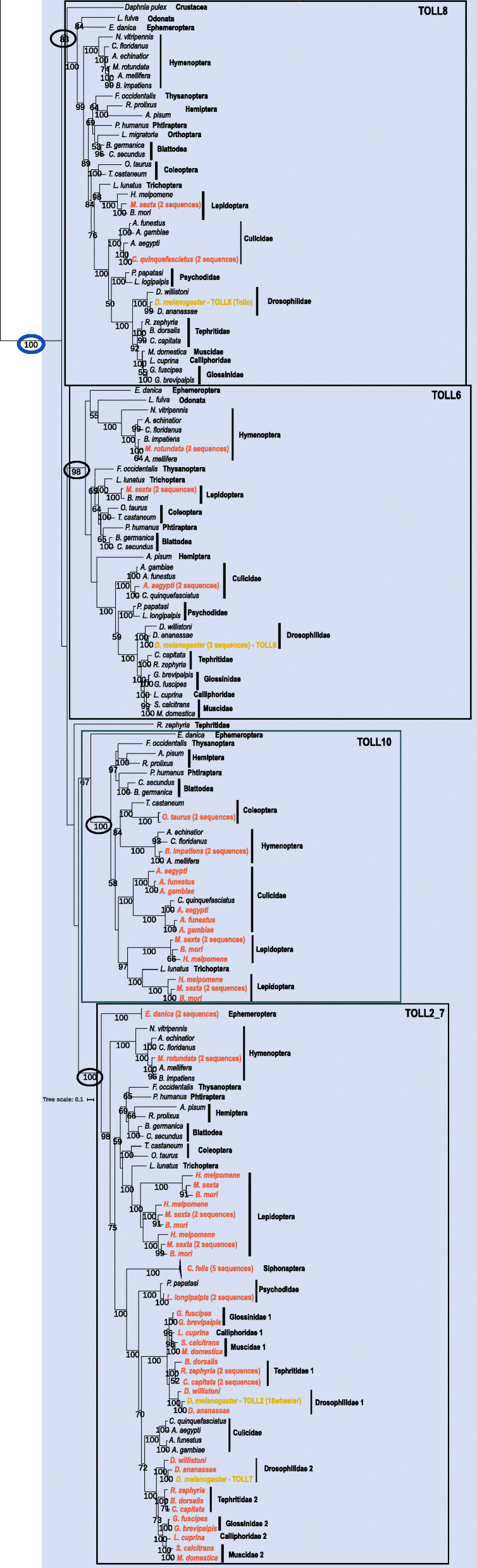
Maximum likelihood phylogeny of the blue clade of TOLL2_7, TOLL6, TOLL8 and TOLL10 proteins. Species with gene duplications are highlighted in orange and *Drosophila melanogaster*’s Toll2, Toll7, Toll6 and Toll8 genes are highlighted on the tree. The black rectangles highlight the clades where *Drosophila*’s genes are found; The gray rectangle highlights the TOLL10 clade. Numbers on branches are bootstrap support values from 1000 replicates and only numbers above 50% are shown. Scale bar is substitutions per site. The image was created using iTOL [84]

Our phylogenetic analyses of the protein alignment of the six gene families of the Toll pathway analyzed here showed very different characteristics (Figs. 2, 3, 4 and 5; (see Additional files 6, 7, 8 and 9)). In all cases, there are duplications within the genomes even though, for the intracellular protein families, the duplications were not as extensive as for Toll and Spatzle (Table 1). For Tube, Pelle, Pellino and MyD88, most species have only one copy of each gene and, when there are duplications, they mostly happened within each taxonomic lineage (see Additional files 6, 7, 8 and 9). When we look at the phylogenetic analysis of Tube (see Additional file 6), we can see that, in Diptera, only *A. aegypti* has two copies of this gene with all other species having only one. The focus in Diptera might have been the reason why most studies cited this and other signal transduction protein families of the Toll pathway as being very conserved [60, 72]. Nevertheless, when we look further to the other insect orders analyzed, another seven had gene duplications (Table 1). At least one Tube gene was found in each genome, including the outgroup *D. pulex* (Table 1 and Additional file 6). The bootstrap values for most interior branches are not high, indicating that there is not enough information within the sequences to confidently infer the relationships among higher taxonomic groups. This might be the reason why the Schizophora Diptera cluster with Hymenoptera instead of with the Culicidae, as was expected [74]. Nevertheless, this is not surprising since the whole insect phylogeny was in debate a few years ago and, as a matter of fact, still is in some points, even though the amount of data used to estimate the relationship of its taxa has greatly increased [3, 74, 78, 85]. One point is certain, within the lineages that have duplications they were species-specific (with high bootstrap support) with gene expansions within each genome (see Additional file 6). To some degree, the same happens in Pelle, Pellino and MyD88, the other signal transduction gene families (Table 1 and Additional files 7, 8 and 9).

In the phylogenetic analysis of Pellino, of the 40 genomes analyzed 17 had gene duplications and at least one gene was found in each genome (Table 1 and Additional file 7). In this case, some of the more basal branches do have high bootstrap values (see Additional file 7) and, apart from two short sequences from *L. fulva* and one from *R. zephyria*, all sequences fall with high bootstrap values within their taxonomic clade. Except for *L. fulva* and *F. occidentalis*, all other duplications, when they occurred, have been within a species genome and bootstrap values are high in each duplication cluster (see Additional file 7). Interestingly, more gene expansions seem to have occurred in the Hymenoptera taxonomic group, with 5 of the 6 species analyzed having more than 2 copies of this gene (Table 1 and Additional file 7). However, this can be an artifact due to the high number of Hymenoptera species analyzed. Both species of Blattodea and Coleoptera analyzed, for example, also have at least two copies of this gene. This indicates that there were more gene expansions in these insect orders than in Diptera, a highly studied group.

In the phylogenetic analysis of Pelle, of the 40 genomes analyzed here nine had gene duplications but, in this case, no proteins were found in eight species even with Exonerate searches (Table 1 and Additional file 8). This is the only gene family analyzed where no genes were found within a species and this might have happened due to the high variability rates found within this protein [72] or, more likely, as discussed above, due to incomplete genome assemblies or gene predictions. This happened in the Hymenoptera, Psychodidae, Tephritidae and Lepidoptera. Again, when duplications did occur, they were clustered with high bootstrap values within a species-specific clade. In the case of MyD88 proteins, of the 40 genomes analyzed here 15 had gene duplications and at least one protein was found in each of the species analyzed, including the outgroup (Table 1 and Additional file 9). All duplications seem to be species-specific with high bootstrap support for these clades, nevertheless, a *B. dorsalis* sequence is found inside Schizophora but outside the Tephritidae clade. Although basal branches do not have high support, apart from Coleoptera and Tephritidae, most taxonomic specific clades do (see Additional file 9).

The phylogenetic analysis of the TIR domain of all Toll sequences retrieved from the species analyzed was able to divide the family into three well supported clades with different evolutionary paths (yellow, green and blue triangles; Fig. 2). All genomes had duplications of Toll genes, with the species *Manduca sexta* having the highest number [28] and a few other species being on the lowest range of five genes (Table 1). Numbers varied widely within taxonomic groups and gene subfamilies (Table 1). The first well supported clade (100% bootstrap) encompasses what we named the TOLL9 subfamily due to the presence of *D. melanogaster*’s Toll9 protein sequences (Yellow group in Fig. 2 and Fig. 3). The clade is further divided into other three well supported clades and, for this subfamily, we can see that in many genomes the gene duplications have occurred sometime in the ancestor lineage of different taxonomic groups. Differently from the other four gene families already analyzed here many were not only species-specific expansions. In *L. fulva*’s genome, for example, there are three different genes, each one belonging to one of the three different TOLL9 clades (Fig. 3). The presence of all three Toll9 genes in an Odonata species suggests that all three genes might have been present in the ancestral Pterygota lineage and one or another have been lost in many taxonomic groups. There are also examples of more recent species-specific duplications with genes from the same genome grouping with high confidence in many cases (Fig. 3). The Coleoptera species *O. taurus* and the Ephemeroptera *E. danica* have the largest gene expansions. This gene is also present in the genome of the outgroup *D. pulex*.

The second highly supported Toll clade (99% bootstrap; green triangle on Fig. 2), contains a few subclades without good bootstrap support in the interior branches (Fig. 4). It includes *D. melanogaster*’s Toll, Toll3, Toll4, and Toll5 genes but, due to the lack of tree resolution, it is difficult to determine which of these, if any, might have been the ancestral gene in Arthropoda. It is clear that all genomes analyzed, even the outgroup *D. pulex*, have at least one copy of this Toll clade, but to which *D. melanogaster* gene other Arthropoda genes are closest it is not possible to say with confidence. Apart from Diptera, in all other species all duplications seem to be species-specific, clustering with high bootstrap values. Nevertheless, for Diptera species, many duplications seem to have happened in an ancestral lineage. The species *R. zephyria*, *C. capitata* and *B. dorsalis*, for example, have a few duplications that seem to have originated in the ancestral lineage of Tephritidae. The TOLL subfamily (where we find the original Toll gene described for *D. melanogaster*) seems to be specific to Schizophora; this Diptera-specific clade has high bootstrap support (95%, black line rectangle in Fig. 4).

The third clade with high bootstrap (100%; blue triangle in Fig. 2) is composed of four subclades with high bootstrap values (Fig. 5). The first subclade was named TOLL8 (83% bootstrap; Fig. 5) due to the presence of *D. melanogaster*’s Toll8 (also called Tollo) gene. The genes in this clade seem very conserved and, apart from *M. sexta* (two identical copies), *C. quinquefasciatus* (two copies) and *C. felis* (not found), most species have only one copy of this gene. The outgroup *D. pulex*, has one TOLL8 subfamily sequence, indicating that this gene was present in the Pancrustacea ancestral lineage. The second subclade was named TOLL6 (98% bootstrap; Fig. 5) due to the presence of *D. melanogaster*’s Toll6 gene. This also seems a very conservative Toll subfamily with most species having only one gene and duplications occurring in only four of the genomes (*A. aegypti*, *M. rotunda*, *M. sexta* and *D. melanogaster*; Fig. 5). Again, most genomes seem to have at least one copy of this gene, although it was not found in the outgroup *D. pulex*.

A third subclade was named TOLL2_7 (100% bootstrap in Fig. 5) due to the presence of *D. melanogaster*’s Toll2 (also known as 18wheeler) and Toll7 genes. These genes are only present in Schizophora species and its duplication might have happened in the ancestral lineage of Diptera and, afterwards, one copy was lost in the Psychodidae and Culicidae (100% bootstrap support; Fig. 5). Perhaps, more likely, it could be a duplication that happened in the ancestral Schizophora lineage since low bootstraps (70 and 72%) are found in the interior branches. Since these genes are an innovation in Diptera, it is difficult to say to which, if any, the insect ancestral sequence was more similar to, so we decided to name this subfamily TOLL2_7. The phylogenetic tree clearly suggests that duplications have also occurred in the ancestral lineage of the Lepidoptera (100% bootstrap support; Fig. 5), with three distinct clusters of *H. melpomene*, *M. sexta* and *B. mori* sequences. The outgroup *D. pulex* is not present in this clade. The fourth subclade has a high support without the *E. danica* sequence (100% bootstrap; Fig. 5) but a lower one if we include this species (67% bootstrap support). It is an interesting clade with only Culicidae species representing the order Diptera. Since no known *D. melanogaster* gene is present, we decided to name it TOLL10, following *D. melanogaster*’s nomenclature. In this clade there were gene duplications in the genomes of *O. taurus* and *B. impatiens* and lineage specific duplications in the Culicidae and Lepidoptera. One *R. zephyria* sequence does not group with high support anywhere in the Blue clade. This might be because its sequence is highly divergent or because it’s genome assembly and gene prediction are not good. Problems with genome assembly and gene prediction can be an issue [86], especially when a large number of highly divergent species are comparatively analyzed.

## Discussion

In this work we evaluated the diversity of Toll pathway gene families in 39 Arthropod genomes, encompassing 13 different Insect Orders, using *D. pulex* as an outgroup. Combining the phylogenetic, domain and residue analysis our data indicates that: 1) As suggested before, intracellular proteins of the Toll pathway have fewer gene duplication events, and we found here that when they happened, they usually are species-specific with important implications for the functional characterization of these genes; 2) we also found that not all Tolls are created equal, and the different Toll subfamilies seem to have different evolutionary backgrounds; 3) the different patterns of gene expansion observed in the Toll phylogenetic tree indicate that homology based methods of functional inference might not be accurate for some subfamilies (such as TOLL, TOLL2_7 and TOLL10); 4) the Spatzle subfamilies are highly divergent and should not be analyzed together in the same phylogenetic framework as has been done previously; 5) network analyses seem to be a good first step in inferring functional groups in these cases. We were also able to see that Toll9 was lost in the ancestral lineage leading to Hymenoptera, and, as suggested before, Toll9 forms a separate subgroup within the Toll family. Moreover, we show that the other Toll subfamilies can also be clustered into other two highly supported clades, where Toll, Toll3, Toll4, Toll5 form a subfamily with more lineage specific expansions in Diptera, whereas the third subclade formed of Toll8, Toll6, Toll2_7 and Toll10 gene subfamilies, seems more conserved. Toll seems to be specific to Schizophora and Toll3, Toll4 and Toll5 are all clustered in Diptera clades making it difficult to estimate which, if any, is the ancestral gene in insects. The presence of a *D. pulex* sequence indicates that Toll8 might have been present in the Pancrustacea, but Toll6, Toll2_7 and Toll10 seem to be Pterygota specific. To our knowledge this is the first work to show, in a phylogenetic framework, that the evolutionary backgrounds of the different Toll pathway genes of the signaling cascade are very diverse suggesting that, particularly in some Toll subfamilies, there might exist different functions in the different insect lineages. Especially important is how this work shows that understanding *Drosophila*’s Toll functions might not lead to the discovery of the same function in other species, even in other Diptera species. We show here how some Toll subfamilies are indeed extremely conserved, but others might have novel duplications which can lead to novel protein functions in specific lineages.

### Evolution of the intracytoplasmic gene families

Studies that analyzed the different gene families involved in the fruit fly and mosquito immune system showed that there might be more gene duplications in the recognition and effector gene families when compared to those that participate in the different signaling cascades. Some variation in copy number has been reported for Toll and Spatzle [60, 71, 72, 87], however, when intracellular members of the Toll pathway are regarded, only 1:1 orthologues have been described [60, 72, 88]. The presence of homologues of all these proteins in vertebrates indicates that this pathway is an ancient and efficient one [18, 28, 89]. Indeed, the presence of sequences of all four intracellular proteins in *D. pulex*’s genome found here indicates that the genes were already present in the ancestral lineage to Pancrustacea. Nevertheless, modifications of the canonical pathway and the number of different functions it can perform already indicates great versatility [29, 38, 90].

Most genomic studies of the intracytoplasmic insect proteins have been done using Diptera species, with only a few including different orders [50, 57, 59, 60, 72, 88, 91–93]. This bias has hidden some copy number variation among insect genomes. In this study, of the 39 insect genomes analyzed here, nine genomes have Pelle species-specific expansions, eight genomes for Tube, 17 genomes for Pellino and 11 genomes for MyD88 (Table 1 and Additional files 6, 7, 8 and 9). The presence of these gene duplications suggests new functions might be present in some species. After a duplication event, the gene copies can follow three main evolutionary paths [77, 94]: 1) neofuctionalization, where the new copy gains a new function; 2) subfunctionalization where the new copy can either split the same function with the ancestral one or even have the same function but in a different cell/body compartment or time in development; 3) or become a pseudogene. Gene duplication followed by subfunctionalization or neofunctionalization has been proposed as an important drive in the evolution of new gene functions [77, 94–97]. In the evolution of the NOX gene family, for example, gene duplication followed by neofunctionalization happened very early in metazoan evolution since both the ability to produce superoxide and hydrogen peroxide were present in the ancestral calcium binding enzymes [98]. The importance of subfunctionalization in gene evolution has also been demonstrated. The vertebrate NOX gene family also has examples of subfunctionalization, where NOX2 seems to be expressed mostly in phagocytes whilst NOX1, NOX3 and NOX4 have other specific functions and patterns of subcellular localization and tissue distribution [99–101].

The Toll pathway was first described as part of the dorsal ventral patterning in *Drosophila*’s development and, since then, many other developmental functions have been found [29]. During development, the perfect expression of genes at the right time and cell/body compartment is important and, in this light, the presence of species-specific duplications in Toll pathway genes might be of significance since these expansions observed here could mean neo or subfunctionalization events. Also, it has been proposed that morphological innovations can be based on differences of timing and location of expression and rewiring of existing gene networks or assembly of new networks with the developmental genes already present [102, 103]. Although most genomics studies so far have reported only one MyD88, Tube, Pelle and Pellino gene copy for insects [57, 59, 60, 71–73, 87, 91–93, 104, 105], in this study, possibly due to the newer genome predictions and the thorough search methods, more genes were found, even for some well-studied species (Table 1). Two copies of MyD88, for example, have also been found in the hemipteran, *Nilaparvata lugens* [106]. The new duplications seen here for many species and in elsewhere [106] may be redundant copies or be part of specific Toll signaling pathways, even of those that have not yet been described, since most occur in understudied species. More than one copy of Tube and Pelle were also found in other arthropod groups such as chelicerates (*Ixodes scapularis*) [71] and many Crustacea (Amphipoda, Isopoda and Decapoda) [107]. The presence of two copies of Pelle in Chelicerata and Crustacea and only one in Diptera lead Lai and Aboobaker [107] to speculate that this duplication might have occurred in the ancestral lineage to arthropods and was later lost in insects. As we can see here some insect species do have more than one copy of this gene, something that would give more support to their hypothesis. Nevertheless, in a phylogenetic framework it is possible to see here that almost all duplications fall, with high confidence, in species-specific clades and, therefore, do not indicate that the copies were present in an ancestral lineage.

An obvious insufficiency or excess of gene expansions among immune genes may reflect different selective pressures from different life environments derived from the diverse ecological niches of insects [60]. Here we analyze 13 insect orders, including holometabola and hemimetabola groups as well as species with diverse lifestyles such as blood feeding habits, for example; making an ideal background for comparing Toll pathway genes. The differences in gene expansion among the genomes can also be due to differences in development, an important function of Toll pathways, or ecological niche, responsible for which pathogens these species might encounter. With species-specific gene expansions, though, one can also argue that these duplication events might have happened very recently by chance and the gene copies could be on their way to becoming pseudogenes. If this is the case with some or all of these gene families’ duplications it can only be determined through experimental laboratory work or population genomics analyses, where we can infer the role of the different evolutionary forces acting on these genes. If gene imbalance is not an issue here and the new copies are neutral, it is quite possible that there hasn’t been enough time for these genes to be lost through random genetic drift. However, comparative analyses of gene losses according to gene ontology (GO) categories have shown that the differences in dispensability observed between different genes might not be stochastic. GO terms related to signal transduction, immune response and other functions that are more sensitive to dosage imbalance are more prone to being lost [108]. These GO terms are less likely to be under the influence of random genetic drift and much more possibly to be under some kind of purifying or positive selection. Moreover, with duplications that have occurred in an ancestral lineage such as the ones found in some Toll subfamilies it is more likely that neofunctionalization or subfunctionalization events might have occurred.

### Evolution of TOLL gene family

The time frame of insect lineage radiations is extremely old and, if dosage imbalance is not an issue, enough time would have passed for genetic drift to expunge or fix any neutral duplications that might have happened in the ancestral lineage of the different taxonomic groups. Hexapods first appeared 479 million years ago (MYA) and important radiation events seem to have happened due to ecologically driven differentiation greatly correlated to major developmental innovations [2, 109, 110]. In the Diptera’s evolutionary history, for example, three main bursts of radiation events happened. In the first, 220 MYA, the ancestral lineages to Psychodidae and Culicidae first appeared, the second one then happened 180 MYA and, finally, a third one was 65 MYA, when most Schizophora lineages appeared [111]. The timing of these bursts of rapid radiations were so brief that in a phylogenetic framework the internal branches are usually very short due to limited information regarding the common evolutionary history of the different lineages. In many cases, such as the Holometabola lineages and other insect orders, internal branches also have higher amino acid substitution rates, further complicating matters [109]. This complicates analyses of ancient gene families where duplication events happened in the ancestral lineages of the groups being studied. The family of Toll receptors has an ancient evolutionary origin, 700 million years ago, being present in different metazoan groups [10, 20, 23, 24, 112]. Arthropod Tolls are homologous to vertebrate TLRs as both are transmembrane proteins with LRRs and a cytosolic TIR domain [23] and play an important role in the immune response in both taxonomic groups. Even though the signaling cascade is very similar with conserved homologues in arthropods and vertebrates, the number of TLRs in each group spans from two unrelated gene expansion events [23, 51, 71]. Arthropod Tolls also do not seem to function as pattern recognition proteins as vertebrate TLRs do [113]. However, a report suggests that Toll7 does bind to VSV virus and induces antiviral autophagy through a noncanonical Toll pathway [114].

While many insect immunity studies have already shown that Toll copy number variation is common among different insect species [72, 87], the different evolutionary paths that each Toll subfamily seems to have been through were not discussed. In this study we found three major Toll phylogenetic clades that correspond to Toll subfamilies with distinctive evolutionary backgrounds that have not been discussed before (Fig. 2). Different studies have demonstrated the closer relationship of Arthropoda Toll9 genes with vertebrate TLRs [71, 72, 115, 116]. Indeed, here the vertebrate-like Toll9 subfamily forms a highly supported clade and is the only insect subfamily where, like vertebrate TLRs, a single cysteine cluster is found in the N-terminal region [71, 89]. The Toll9 clade is further subdivided into three well supported clades (Fig. 3) and, interestingly, the presence of 1 *L. fulva* (an Odonata) sequence in each of the three subclades found, suggests that three Toll9 genes might have been present in the Pterygota ancestor. Duplications of Toll9 were also found in the lepidopteran *Plutella xylostella* when its genome was characterized [117]. Although *D. pulex* is only found in the first subclade, Blattodea, Orthoptera and Lepidoptera sequences can be found in two subclades, whilst species from the other orders, including Diptera are only found in one. Since most insect studies have encompassed Diptera and only a few species from other orders, this subclade subdivision was missed in earlier studies. Due to the ancient evolutionary history of Tolls and the pattern of gene loss found for Toll9 here, where some orders are found only in different subclades, it is quite possible that three different genes were present in the ancestral lineage and some or all were differentially lost in some lineages. This subfamily shows lineage as well as species-specific expansions. In Diptera for example, most gene expansions are species-specific but in Culicidae there was a duplication that happened in the ancestral lineage leading to the *Culex* and *Aedes* genera. Big species-specific expansions happened in the Ephemeroptera *E. danica* and the Coleoptera *O. taurus*. Immunity related genes are frequently under the birth and death evolutionary model [118], where new genes are formed from gene duplications and others are lost due to the accumulation of deleterious mutations. This dynamic of gene gain and loss, in some cases, seems to be influenced by adaptation to new ecological niches and physiological changes during evolution [109], something that has happened many times in insect evolution.

In Hymenoptera no genes were found that belonged to the Toll9 subfamily. The evidence for gene loss is negative and can pass unnoticed or not be considered due to uncertainties in the completion or assembly of sequenced genomes. This can certainly be the case with Trichoptera, where only one species was analyzed and no Toll9 gene was found either. Nevertheless, six species of Hymenoptera belonging to different taxonomic families were analyzed here. The impact of gene loss in the evolution and function of surviving paralogues is not well investigated. It is easier to recognize gene duplication and the appearance of a new gene function as adaptive. However, it is also possible that the loss of a gene function altogether might not have a detrimental or adaptive effect on a species and, in fact, can be neutral [108, 119]. Neutral or nearly neutral gene losses can be fixed in a species through genetic drift. Another possibility is the presence of other genes such as paralogs, analogs or even whole different pathways that serve the same or very similar functions and, therefore, the loss of a specific gene does not mean loss of function (mutation robustness). This can certainly be the case with Toll genes as a gene belonging to another subfamily may well have the same or similar function Toll9 was responsible for previously. The different functions each Toll subfamily gene has are still being discovered and it seems that, in some cases, functions do overlap [50, 120–122]. Toll receptors are an ancient gene family and thus their participation in different biochemical processes and patterns of gene gain and loss are expected. Another good example is the absence of NOX-art genes in Hymenoptera (among other insect orders as well) [123]. NOX is a gene family that first appeared in multicellular animals and, among their many roles, immunity is one of them. Some Hymenoptera species seem to have one order of magnitude higher rates of gene gain/loss than other insects mostly sprouting from single gene gain/loss in a large number of gene families [109].

In agreement with Palmer and Jiggins [71], that studied Toll genes in different arthropod lineages, no 1:1 orthologue was found for Toll (or Toll1) subfamily genes outside of Schizophora (Fig. 4). As such, and as seen for other arthropods [71], it is not possible to infer which Toll subfamily is responsible for immunity functions in other insect species other than Diptera. Also, since interior branches have low bootstrap values, it is difficult to say which, if any, of the other *Drosophila*’s Tolls present in this clade (Toll3, Toll4 and Toll5) is the ancestral one. The high bootstrap value (100%) suggests that Toll3 and Toll4 duplications happened in the Drosophilidae ancestral lineage (this study) and were lost in different degrees in the different *Drosophila* species [122]. These genes are under positive selective pressure in *D. willinstoni* and it was speculated that they might bind to new ligands other than Spatzle. These two genes seem to have lost their developmental functions since *D. melanogaster* knockdowns have little influence in viability [122]. These subfamilies are good examples of how some of the Toll pathway genes evolve, with duplication followed by positive selection in some cases or pseudonization in others. Other genomic studies of both vertebrates and invertebrates have already shown that Toll receptors have undergone diversification by mechanisms of genetic duplication, neofunctionalization, and subfunctionalization [124, 125]. This diversification is possibly the reason the Toll family of proteins recognize a variety of extracellular and endosomal stimuli, participating in a number of different pathway responses.

In agreement with Levin and Malik [122] the subfamilies Toll8 and Toll6 seem to be conserved, with no lineage specific gene expansions and very few species-specific ones. Although Toll2_7 was also considered a conserved subfamily with > 90% amino acid identity among *Drosophila* species [122], extensive species-specific and lineage-specific expansions can be seen here especially in Lepidoptera and Diptera. The fact that studies so far mostly focused on *Drosophila* species or even Diptera have led to the belief that Toll genes evolve slowly and with little gene turnover (gain/losses), which would be consistent with their important roles in development and immunity. Nevertheless, even in this Toll clade where more conserved subfamilies are found (Toll8 and Toll6), various gene expansions suggest that, in some insect lineages at least, positive selection and new functions may have arisen. Especially for Toll2_7, gene gain and loss has happened on many occasions. A novel finding of this study, the Toll10 clade, seems to have been lost in Schizophora altogether and suggests how interchanging the Toll functions can be. The duplications of Toll2_7 in this lineage or any of the other duplications found in other Toll subfamilies could have the role Toll10 can perform in the other taxa. Although this is certainly possible, the SSN indicates that Toll10 is very dissimilar from other *Drosophila* Tolls and might have a different function (see Additional file 4). The Toll subfamilies phylogenetic tree and its pattern of gene gain/loss found here and in the network analysis (see Additional file 4) indicates that homology-based methods of functional inference might not be accurate. Most functional studies have focused on Diptera so far (especially in *D. melanogaster*) and, therefore, understanding gene function in one species might not lead to discovering the same function in another, not even other Diptera species. Taking into account the evolutionary pattern and the lack of 1:1 orthology in most subfamilies, it is important that experiments with species from different taxonomic groups are performed in order to better understand gene function of the different Toll genes.

### Evolution of Spatzle gene family

Although high divergence is found among the different Spatzle subfamilies, SSN analysis indicates that only for Spz1 and Spz7 an homology-based functional inference might be problematic. Even though a phylogenetic framework was not possible, network analysis shows that Spz1 is composed of a few taxa-specific iso-functional groups and Spz7 does not have any Diptera species and, therefore, no previously characterized functions (Fig. 1) (see also Additional file 5). A characteristic intron-exon structure found within a cysteine-knot in Spz, Spz2, Spz3, Spz5 genes suggests that these genes may have arisen by gene duplication events [55]. The presence of the cysteine residues needed for the 3D structure formation indicates that the *Drosophila*’s homologues of Spatzle can also be activating ligands for Toll receptors [55, 126] and many different Toll/Spz interactions have also been demonstrated in the lab [90]. Nevertheless, the primary sequences of each Spatzle homolog found in *Drosophila*, and other insect species, are highly divergent. Within the different subfamilies, protein sequences show some degree of conservation (70–90% sequence similarity among *Drosophila* sequences) [126], nevertheless, among the different subfamilies high divergence is observed (20–40%) [55, 126]. Not surprisingly, previous works that have used distance tree building methods to better understand the evolution of the different Spatzle homologues in a few insect species have found low bootstrap support in internal branches [91, 126, 127].

Due to the high number of different species analyzed here, the low sequence similarity found among the Spatzle subfamilies prevented a phylogenetic reconstruction (< 30%), nevertheless, the SSN seemed to work well in defining the iso-functional groups (Fig. 1). Although this is not an evolutionary approach since it does not reconstruct the historical relationships among the sequences [79, 128], the groups recovered here are in agreement with the distance trees already published using fewer insect species [91, 126, 127]. The presence of sequences from most taxonomic groups within the iso-functional clusters in the SSN suggests that the duplication events that formed this protein family might have happened in the ancestral Pancrustacea, or multiple events of convergent evolution must have happened. Most *D. pulex*’s sequences are highly divergent and compose a separate functional group but there are also sequences in the Spz3, Spz5, Spz6 and the new Spz7 cluster. Indeed, Wang and Zhu [126] analyzing only five insect orders (20 insect species, 12 of which were *Drosophila*) found 1:1 orthologues for each Spatzle subfamily. The downside to SSN is that the distinction between paralogues and orthologues is not possible [129] rendering it not feasible to determine if sequences have originated through species- or lineage-specific expansions.

Although network analysis only shows similarity between sequences it does use an amino acid substitution matrix model to compute these similarities and within the iso-functional groups one could argue that multiple substitutions are not an issue. In fact, this methodology is being used to help annotate uncharacterized proteins using identity levels with sequences of known function [80, 130]. However, this methodology needs to be used with caution since paralogues that belong to the same iso-functional group might still have gone through subfunctionalization or another evolutionary process that renders them a different function albeit having low divergence. Our SSN analysis corroborates the trees constructed with distance algorithms, with Spz1 subfamily being more diverse (longer branches and low branch support) and with Spz3 and Spz4 subfamilies having higher similarity [91, 126, 127]. The SSN shows Spz3 and Spz4 belonging to the same functional group and Spz1 sequences in a less cohesive cluster, with Diptera species having lower identity values when compared to other insects (Fig. 1). Indeed, *Drosophila* Spz3 and Spz4 sequences have greater identity (51% similarity) when compared to sequences from the other subfamilies (20 to 38%) [55]. In this study a new functional group composed of species from many taxonomic groups but without Diptera was also formed (Fig. 1) (see also Additional file 5) suggesting a previously unknown iso-functional group that might have been lost in Diptera. Although Sequence Similarity Networks (SSN) are not the same as a phylogenetic analysis it seems to work well for identifying functional groups within highly divergent protein families such as Spatzle as was demonstrated before for other families [128, 131, 132].

## Conclusion

The increased number of available genomes is facilitating gene content and evolutionary analysis of many gene families. It is believed that only circa 50% of the proteins discovered through genome sequencing projects have correct functional annotation [133]. Here we analyzed different aspects of some protein families involved in the Toll pathway and, through computational methods, shed some light into their evolution and functional annotation. Our results show the importance of using as many species as possible, representing the different insect orders, to better understand gene content, origin and evolution in insects. The gene families analyzed in this study have an array of developmental and immune roles and the interaction between the different proteins seems to be of significance in the role they play. The joint use of phylogenetic and network methods works well, especially when highly divergent protein sequences are present within a family. The evolutionary patterns of many protein subfamilies found here indicate that homology-based methods of functional annotation might not be reliable in many cases. In a group as diverse and ancient as insects, phylogenetic and/or SSN investigations are necessary to better understand the different functional groups within a protein family. Our results suggest there is a subclade division within TOLL9 with three different genes that were probably present in the ancestral Pterygota lineage, with different patterns of gene gain and loss among the insect orders. We also show that the original Toll gene described and the TOLL2_7 clade are Diptera specific, indicating that functional inference from Diptera functional studies might not be readily transferable to species from other Orders. A new TOLL10 subclade with no *Drosophila* sequences was identified and we clearly show that the different TOLL subfamilies have divergent evolutionary histories that should be taken into consideration. We also show that events of gene gain in the intracellular gene families is pervasive and they mostly occur through species-specific expansions, suggesting the appearance of new functions in different species. The high number of insect species [39] analyzed here meant that species of public health and ecological importance were also investigated, and this can be of assistance when choosing specific genes to be used in new methodologies for pest and vector control. Finer, more species-specific methods of pest and vector control are essential to lessen the ecological impacts and health hazards these interventions usually have.

## Methods

### Protein searches

To search for all proteins involved in the Toll signaling pathway in the 40 arthropod genomes analyzed (39 Hexapoda and the Crustacea *Daphnia pulex*, Table 1), Hidden Markov Model (HMM) Profiles of each gene family were used as queries in HMMsearches [134] on the predicted protein sets using the FAT pipeline (developed by RD Mesquita). Profiles were retrieved from the Pfam database [135, 136]. For Toll and MyD88, the Toll/interleukin-1 receptor (TIR) homology domain (HMM profile PF01582) was used. For Tube, the Tube Death Domain (DEATH_2) (PF14786) was used, and for Pelle both the protein kinase (Pkinase) and Death (DD) domains (PF00069 and PF00531) were used. Spatzle (PF16077) and Pellino (PF04710) domains were also used as queries. All proteins with significant E-value (< 0.001) were retrieved and used as queries on BLASTp searches [137] against the manually curated Uniprot/SwissProt protein database [138, 139], also using FAT. Since the sequences were searched on the predicted protein databases, whenever a specific protein was missing from a genome, Exonerate (protein2genome mode) [140] searches against the scaffolds of the whole genome were performed. For this step, the already predicted protein from the closest species available was used as query with the command line: exonerate -m p2g --showtargetgff -q ortologos.fasta -t scaffolds.fasta --ryo “>%ti (%tab-%tae) predicted by %qi Strand %g ID= %pi Positives= %ps Raw_score= %s \n%tcs\n” > output_exonerate.txt. With these settings we could retrieve the coding sequences (CDS) of the genes searched, in the first reading frame, and then the newly predicted gene was translated using Transeq [141]. This search ensured that we could find genes that were not automatically predicted when they were present in the genomes. Also, partial gene sequences were also used as queries in tBLASTn searches against the NCBI Transcriptome Shotgun Assembly (TSA) database [142] in an attempt to retrieve a complete peptide for the subsequent phylogenetic analysis. The sequences of all peptides found and their accession numbers are available for download in FASTA format (see Additional files 10, 11, 12, 13, 14 and 15).

### Domain analysis

The TIR domain is present both on Toll-like receptors and other signaling proteins [51, 113, 143]. In arthropods, it is also present in MyD88, an adaptor protein that participates in the Toll cascade, thus the HMMsearches were performed concomitantly. Nevertheless, to further investigate these two different proteins and work with genes with the same evolutionary background in further analysis, a characterization of the different domains in each protein was warranted. Also, for the protein kinase Pelle the search retrieved many non-specific kinases that needed to be analyzed and the domain analysis helped sift through the HMM and BLASTp results. In any case, all proteins with significant E-values had their domain structure predicted using the CD-search batch tool [144, 145] and SMART [146, 147]. Transmembrane helices and subcellular localization were predicted with TMHMM and TargetP [148], respectively. Among the proteins found, only those with the expected domains, regions and sizes were used in the phylogenetic analyses.

### Sequence similarity networks (SSNs)

The collection of protein sequences extracted with TIR and Spatzle Pfam domains from all 40 genomes analyzed were used to construct sequence similarity networks (SSNs). SSN is a methodology used to summarize protein-protein similarities on a large scale [80, 81] and, therefore, we decided to first investigate all proteins obtained with these domains with this methodology. Especially for the TIR domain, where it was expected that more than one functional protein family could have been found on the HMMsearches, the use of the SSN made it possible to separate the different functional and orthologous groups. Also, with this methodology, sequences that could not be used in the multiple alignments and phylogenetic analyses due to small sizes and incomplete prediction could be investigated as well since this is mainly an all-by-all BLASTp pairwise sequence analysis [79, 80]. Although not a global alignment, newer versions of BLAST do extend the alignment and take gaps into account when computing alignment scores. On top of the two Pfam domains, we also used a smaller sequence set of only Toll-like proteins to better understand the functional groups within this protein family. The complete protein sequences were used in all three SSNs and different threshold values were tested for each protein set. SSNs have been shown to work well in identifying functional groups and revealing outliers [79]. The Enzyme Function Initiative - Enzyme Similarity Tool [80, 81, 131, 149] was used to cluster the sequences using user defined similarity thresholds. Here each node in the network represents proteins with 100% sequence identity and the edges represent the similarity between the nodes. The threshold defines the number of edges since an edge is drawn between nodes only if the BLAST pairwise similarity score between them is above the threshold value defined. The alignment score (threshold) used to restrict the all-by-all BLASTs in all three networks varied. Differently from a normal BLAST result, EFI-EST alignment score is not dependent on database size [80]. For each gene family it was chosen based on the percent identity versus alignment score quartile plot with a 40% identity threshold as advised by the authors. The SSNs were visualized using the Organic layout (Wiese yFiles) in Cytoscape 3.6.1 [150] where relevant information for each sequence was also mapped (taxonomy, Swissprot results, domain information, functional annotation). OrthoMCL 1.4 [151] results with a cut off *P*-value of 1E-05 were also used as a guide for Spatzle orthologous group formation.

### Phylogenetic analysis

Amino acid sequences of the proteins retrieved by our searches were aligned locally with PASTA [152] using mafft [153] and a Jones Taylor Thornton (JTT) matrix [154]. The alignments were visualized and converted to Phylip format using the software SeaView [155]. The same program was used to trim the sequences leaving only the region containing the TIR domain for Toll proteins. This way, the variable regions containing the cysteine knots and LRR were eliminated from the alignment. This trimmed version of the alignment for Toll and the whole alignment for MyD88, Tube, Pelle and Pellino were then used to construct phylogenetic trees for each gene family using the maximum likelihood method with RAxML [156] on CIPRES [157]. The amino acid JTT scoring matrix was used [154] and bootstrap analysis with 1000 replicates was performed to infer branch support. Visualization and further editing of the trees was performed on the web tool iTOL [84]. Since Hexapoda is phylogenetically closer to Crustacea [1, 74], more specifically to the Branchiopoda [158], and the most recent Arthropoda Toll pathway evolutionary gene study has used *D. pulex* in their analysis [71] we decided to use this species as the outgroup.

## Supplementary Information

**Additional file 1.** List of genes found with Exonerate. Species name, scaffold and genomic region where they were found and if they were used in the phylogenetic analyses.

**Additional file 2.** Text file, in FASTA format, with protein sequences found with Exonerate Searches and translated with Transeq.

**Additional file 3.** Figure in TIFF format with the SSN of the TIR domain proteins found on FAT searches. Each node represents proteins sharing 100% sequence similarity and edges represent an alignment score cut-off of 20 between proteins. The nodes are colored based on Toll (magenta) and MyD88 (teal) functional groups.

**Additional file 4. **SSN of only TOLL 1 genes (from Additional file 3). Each node represents proteins sharing 100% sequence similarity. The network was created using an alignment score cut-off of 20 and, in Cytoscape, an identity value of 50% was also used as threshold and edges with lower values were deleted. The nodes are colored based on taxonomic groups (see legend). and edges represent an alignment score cut-off of 20 between proteins. Protein names within the figure depict *Drosophila melanogaster*’s known genes that are represented in triangled shaped nodes.

**Additional file 5. **SSN of the Spatzle domain proteins found on FAT searches. Each node represents proteins sharing 100% sequence similarity and edges with an alignment score cut-off of 30 between proteins. A) Nodes are colored based on taxonomic groups and B) with only *Daphnia pulex* nodes highlighted in green.

**Additional file 6.** Maximum likelihood phylogeny of aligned Tube proteins. The blue square highlights Hexapoda species and the species with gene duplications are highlighted in orange. Numbers on branches are bootstrap support values from 1000 replicates, only numbers above 50% are shown. Scale bar is substitutions per site. The image was created using iTOL.

**Additional file 7.** Maximum likelihood phylogeny of aligned Pellino proteins. The blue square highlights Hexapoda species and the species with gene duplications are highlighted in orange. Numbers on branches are bootstrap support values from 1000 replicates, only numbers above 50% are shown. Scale bar is substitutions per site. The image was created using iTOL.

**Additional file 8.** Maximum likelihood phylogeny of aligned Pelle proteins. The blue square highlights Hexapoda species and the species with gene duplications are highlighted in orange. Numbers on branches are bootstrap support values from 1000 replicates, only numbers above 50% are shown. Scale bar is substitutions per site. The image was created using iTOL.

**Additional file 9.** Maximum likelihood phylogeny of aligned MyD88 proteins. The blue square highlights Hexapoda species and the species with gene duplications are highlighted in orange. Numbers on branches are bootstrap support values from 1000 replicates, only numbers above 50% are shown. Scale bar is substitutions per site. The image was created using iTOL.

**Additional file 10.** Text files, in FASTA format, with protein sequences found with FAT searches of Tube.

**Additional file 11.** Text files, in FASTA format, with protein sequences found with FAT searches of Pellino.

**Additional file 12.** Text files, in FASTA format, with protein sequences found with FAT searches of Pelle.

**Additional file 13.** Text files, in FASTA format, with protein sequences found with FAT searches of MyD88.

**Additional file 14.** Text files, in FASTA format, with protein sequences found with FAT searches of Toll.

**Additional file 15.** Text files, in FASTA format, with protein sequences found with FAT searches of Spatzle.

## Data Availability

All data generated or analyzed during this study are included in this published article [see Additional files 1, 2, 3, 4, 5, 6, 7, 8, 9, 10, 11, 12, 13, 14 and 15). The FASTA files with the sequences used can also be retrieved in https://www.dropbox.com/sh/lkksoj542qn6kqj/AAB3pKV2A38bY2cCtX3i7AJxa?dl=0.
