# Supplementary figures and images for: Evolution of Toll, Spatzle and MyD88 in insects: the problem of the Diptera bias
Source: BMC Genomics. 2021 Jul 21;22:562. doi: 10.1186/s12864-021-07886-7 (PMC8296651; doi:10.1186/s12864-021-07886-7)

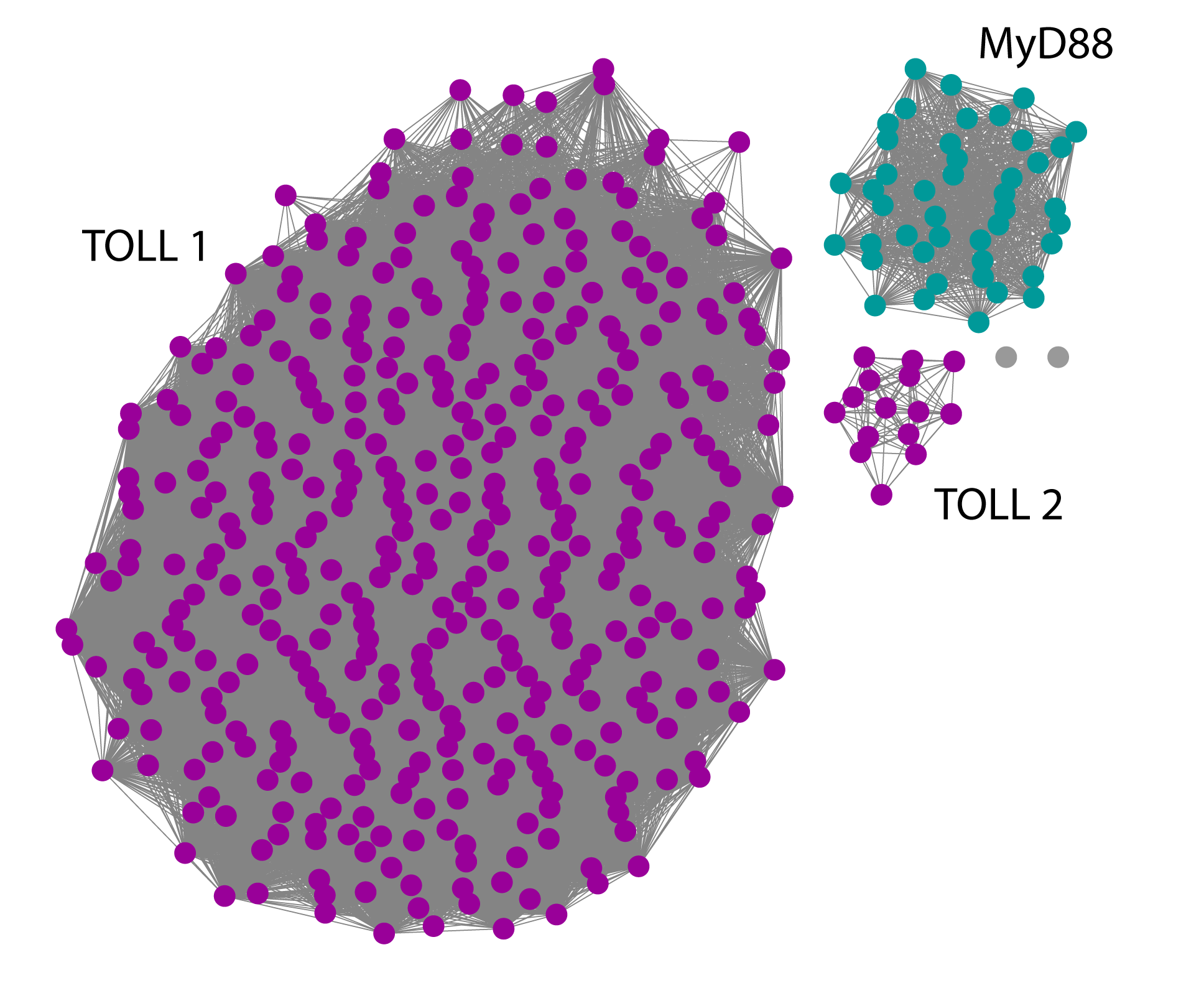

Supplement: Supplementary file 3 — Additional file 3. Figure in TIFF format with the SSN of the TIR domain proteins found on FAT searches. Each node represents proteins sharing 100% sequence similarity and edges represent an alignment score cut-off of 20 between proteins. The nodes are colored based on Toll (magenta) and MyD88 (teal) functional groups. [file 12864_2021_7886_MOESM3_ESM.tif]
